# A Novel Postbiotic Reduces Canine Halitosis

**DOI:** 10.3390/ani15111596

**Published:** 2025-05-29

**Authors:** Aylesse Sordillo, Liza Casella, Raphaël Turcotte, Ravi U. Sheth

**Affiliations:** Kingdom Supercultures, Brooklyn, NY 11205, USA

**Keywords:** canine, halitosis, postbiotic, periodontitis

## Abstract

A majority of dogs suffer from some form of periodontal disease. This frequently manifests as halitosis or oral malodor, caused by microbes underlying poor oral health. Pathogenic oral microbes process dietary or host proteins into volatile sulfur compounds (VSCs), which are perceived as malodorous and can further contribute to inflammation and periodontal disease progression. This double-blind, placebo-controlled, randomized clinical trial evaluated the ability of Superculture^®^ Pet Oral, a novel canine oral health postbiotic (COHP), to reduce canine oral malodor. In total, 24 dogs were stratified into two groups based on starting VSC levels. The groups received either COHP or a placebo as a powder topper for 14 days. VSC levels were measured via a Halimeter on Days 0, 7, and 14. Perceived malodor was also scored on the same days. COHP significantly decreased VSCs from baseline by Day 7 by 22% (*p* = 0.002). In the placebo group, VSCs increased over the course of the study. Throughout the study, COHP lowered VSC levels by 27% compared to the placebo (*p* = 0.004), and fully prevented an increase in VSCs compared to the baseline. Additionally, VSCs were correlated with the human perception of malodor, and twice as many dogs in the COHP group had perceptibly improved breath on Day 7 compared to the placebo, measured through a 10-point scaled survey. These findings validate the ability of this novel postbiotic to effectively reduce canine oral malodor and provide preliminary evidence that it may more broadly help to maintain canine oral health.

## Simple Summary

Dental disease is a prevalent issue in dogs and is driven by the oral microbiome. Specific pathogenic microbes in the oral cavity produce volatile sulfur compounds (VSCs); these VSCs are the causative agents of malodor (bad breath) and are more broadly associated with poor oral health. Reducing canine oral malodor is not only a consumer benefit but is important in maintaining canine oral health. The efficacy of existing natural ingredients is limited, and there are health risks and downsides associated with chemical actives. In a double-blind, randomized clinical trial, a novel postbiotic oral health ingredient, Superculture^®^ Pet Oral, was able to reduce the compounds responsible for canine oral malodor within seven days and lower these compounds by 27% compared to placebo group. These results indicate that this postbiotic can provide a practical and effective solution to a widespread health issue in the canine population by impacting oral microbiome function.

## 1. Introduction

Halitosis, commonly known as bad breath, is a condition characterized by unpleasant odor emanating from the oral cavity [1]. In dogs, as well as in humans, halitosis is primarily caused by specific microbes, which can break down sulfur-containing amino acids such as cysteine and methionine to release VSCs, primarily hydrogen sulfide (H_2_S) and methyl mercaptan (CH_3_SH) [2,3,4]. These compounds are perceived as offensive by humans [5,6]. Studies suggest that halitosis is prevalent in dogs, affecting a significant portion of the canine population, with estimates indicating that up to 80% of dogs over the age of three suffer from some form of dental disease [7,8].

There is a strong link between halitosis and periodontal disease, as the buildup of plaque and tartar from bacteria leads to further bacterial proliferation, contributing to bad breath [9,10,11]. Periodontal diseases in dogs include gingivitis, the inflammation of the gums, and periodontitis, a more severe condition that involves the destruction of the supporting structures of the teeth [12]. In vitro evidence suggests that VSCs, especially H_2_S, can also promote the progression and increase the severity of periodontal diseases [13]. H_2_S exposure increases apoptosis of gingival fibroblasts [14] and epithelial cells [15], and promotes inflammation through the release of pro-inflammatory cytokines [16,17,18]. CH_3_SH exposure promotes the secretion of factors involved in tissue degradation [19] and increases the breakdown of collagen [20] by gingival fibroblasts. Both compounds have been shown to increase the permeability of the oral mucosa [21]. These pathways contribute to the pathogenesis and severity of periodontitis [22]. In addition to improving social interactions between dogs and humans, maintaining good oral health is therefore essential in preventing periodontal diseases, which can be irreversible and are associated with serious, systemic health problems such as cancer, endocarditis, and inflammatory diseases [23,24].

While daily tooth brushing and professional cleanings are recommended, low adoption by owners necessitates alternative preventative solutions. It is recommended that dog owners brush their dogs’ teeth daily to remove plaque, prevent tartar buildup, and avoid halitosis [8,25,26]. However, many dog owners face challenges with this practice due to their pets’ uncooperative behavior or practical difficulties [7,8,27]. Professional dental cleaning by a veterinary dentist may be necessary to thoroughly clean teeth both above and below the gumline. Unlike in humans, this procedure requires general anesthesia in dogs, which increases the cost and carries inherent risks [28,29]. A study found that only 13% of dogs had undergone professional dental cleaning under anesthesia, highlighting the limited utilization of this service [8].

Existing solutions that have a higher adoption rate due to their ease of use are limited in their efficacy, particularly when it comes to effectively treating the underlying microbial causes of poor oral health. There is limited evidence that herbal remedies and essential oils are efficacious in canines, and they are often evaluated in addition to other solutions [30,31,32]. In humans, they have been found to be less effective than chemical actives and have failed to provide additional benefits when compared to conventional toothpaste [33,34]. Providing dental chews can, through mechanical cleaning action, remove plaque [35,36]. It is commonly understood that these chews target supragingival plaque, which is standardly assessed. However, some recent studies have demonstrated that dental chews can affect the subgingival microbiome [37,38]. Chemical ingredients, such as sodium hexametaphosphate (SHMP), target a wide array of oral microbes, including potentially desirable microbes that support a healthy oral microbiome [39]. SHMP functions at least partially by binding to calcium ions in the saliva, thereby preventing the mineralization of plaque into tartar [40], but can also cause adverse effects. Dogs treated with a mixture of three polyphosphates, including SHMP, in short-term studies experienced weight loss, decreased kidney function, and increased heart weight (hypertrophy) [41]. While this short-term study used relatively high doses of polyphosphates (1–4 g/kg/day), additional long-term studies in rodents suggest that chronic exposure to low doses of SHMP (as low as 0.1–0.5% inclusion in feed) could lead to adverse effects, making it unideal for regular oral care [41]. Other natural ingredients, such as brown algae (e.g., *Ascophyllum nodosum*) are believed to have systemic effects that may reduce plaque and calculus accumulation, but the exact mechanism is unclear and the overall efficacy of brown algae is variable and greatly affected by form factor [42,43]. Often, mechanical chews include other active ingredients that are meant to more effectively treat microbial pathogens [44,45,46,47,48]. However, in studies that compare chews with and without additives, head-to-head evidence of improved efficacy is limited [44,47,48].

Microbially derived ingredients show great potential at improving overall dental health [49]. Beneficial oral bacteria produce antimicrobial peptides and other metabolites known to inhibit periodontal pathogens associated with oral malodor [50,51,52]. However, probiotics, which contain live cultures, face efficacy challenges as they lack stability under the challenging temperature, pressure, and moisture constraints associated with the manufacturing and storage of pet food, treats, and supplements as well as the pH range and enzymes encountered in the oral cavity [49,53,54].

Postbiotics are microbially derived ingredients that provide a promising alternative by which to impact the oral microbiome function. A postbiotic is an inanimate probiotic and its metabolite constituents that confers a health benefit on the host [55]. Thus, they contain microbial products, which may allow them to impact pathogenic microbes more specifically and efficaciously than the alternative solutions described, and they are also more stable than probiotics and can be incorporated into a range of products. Indeed, postbiotics have been shown to provide oral health benefits in in vitro, mouse, and human models. A bioactive metabolite generated by microbes during fatty acid metabolism repaired *Porphyromonas gingivalis* induced damage to gingival epithelial cells in vitro and suppressed the bacteria-induced degradation of E-cadherin and subsequent inflammatory cytokine production in the gingival tissue of mice [56]. In humans, postbiotic lozenges caused a trend toward a decrease in VSC levels and decreased pathogenic microbes associated with VSCs [57]. Further testing of the same postbiotic showed it decreased VSC levels in vitro and that compounds present in the postbiotic had the ability to decrease oral biofilms and VSCs in vitro [58]. However, there is very limited clinical evidence of their effectiveness at reducing halitosis in dogs.

In this work, we evaluated the potential of a novel canine oral health postbiotic (COHP) in reducing oral malodor in dogs in a double-blind, placebo-controlled, randomized clinical trial.

## 2. Materials and Methods

### 2.1. Animals

The study was conducted at a registered research facility that complied with all local regulations governing the care and use of laboratory animals and was conducted in accordance with OMAFRA, the CCAC Guide to the Care and Use of Experimental Animals. To ensure compliance, the protocol was reviewed and approved by the facility’s Institutional Care and Use Committee (IACUC).

All dogs were part of a permanent colony made up of beagles and small mixed breed dogs. All participants were between 1 and 7 years old. Both intact and neutered animals of both sexes were included in the study. Dogs were allowed to socialize in groups in outside dog runs for at least one hour each day and had access to a larger dog park at least twice weekly for robust play and exercise.

All dogs were either pair-housed in 10-foot × 10-foot runs that can be divided into 5-foot × 10-foot runs for individual housing or group-housed in 6.1 m × 4.9 m rooms, with 6–8 dogs per room. Beds and blankets were provided to all dogs. Fresh, clean, drinking water was provided ad libitum, but was withheld during feeding (~30 min/day). Dogs remained in the same room for the duration of the study. Animal rooms were cleaned at least once daily, disinfected twice weekly, and descaled when needed.

All dogs are classified as Category C for the full duration of the study: animal use activities that involve no more than momentary or slight pain or distress for which there is no need for use of pain-relieving drugs.

The inclusion criteria in the study were as follows: (1) participants were not taking any prescribed medications or only prescribed NSAIDs or routine medication for fleas, ticks, or heartworm, (2) participants’ body condition scores were between 2 and 8, and (3) participants were not pregnant and had not been pregnant in the last 6 months.

### 2.2. Study Design

The study was a double-blind, placebo-controlled, randomized design. The duration of the study was 15 days (Day 0–14), and the treatment was administered for 14 days (Day 1–14). VSC levels were measured at Day 0, and two groups of 12 dogs each were stratified to be matched by initial VSC levels (Day 0) and sex.

The study used a dirty tooth model (no dental cleaning occurred as part of the study). Prior to the study, there was a 5-day acclimation period, during which the participants did not receive any oral care by a veterinary dentist, did not have their teeth brushed, and did not consume or use any oral health product, including dental chews, treats, and food supplements. No dental chews, treats, or supplements were provided through the duration of the study outside of the treatment. In addition, ad libitum access to routine dental enrichment, such as chew toys and bones that may disrupt plaque via mechanical action, was interrupted for the duration of the study, as is standard in these types of studies. This dental enrichment is the only oral health care regularly provided to the dog colony. All participants had mild to medium halitosis (50–400 ppb VSCs were detected in breath samples as measured experimentally by a Halimeter^®^ PLUS (Interscan Corporation, Camas, WA, USA) on Day 0).

There were two termination criteria for the study: (1) Abnormal changes in a dog’s health as assessed by a veterinarian followed by a recommendation by a veterinarian to remove the dog from the study and (2) refusal to eat more than 2 consecutive meals.

### 2.3. Treatment

COHP is a commercially available ingredient (Superculture^®^ Pet Oral ingredient, Kingdom Supercultures, Brooklyn, NY, USA) composed of a tapioca maltodextrin carrier, dried *Pediococcus pentosaceus* fermentation product, and dried *Bacillus subtilis* fermentation product. Both fermentation products are heat treated to inactivate live cells, then spray or freeze dried. The placebo was tapioca maltodextrin, the same carrier utilized in the ingredient.

On Days 1–14, each dog received 250 milligrams of either COHP or the placebo added to their only meal of the day (standard dry diet; Purina Dog Chow) as a powder topper. The daily food portion was placed into a bowl and sprayed with enough water to moisten the food and promote adhesion of the powder topper to the food. The dog’s water source was removed, the dog was served the food containing the powder topper and given up to 30 min to eat all the food. The dog’s water source was replaced upon completion of the meal and the dog had access to water ad libitum for the rest of the day.

### 2.4. Breath Sampling

After the dog’s meal, a single trained technician, who was different from the breath scoring technician, performed all breath sampling measurements on Days 0, 7, and 14 using the Halimeter^®^ PLUS (Interscan Corporation, Camas, WA, USA) and recorded the VSC level data in a separate spreadsheet from the breath scoring data. The Halimeter was zeroed and calibrated to the ambient air in advance of any measurements. In advance of any measurements, the dog’s mouth was gently held closed for 1 min. The Halimeter probe was inserted between the canine teeth such that the air in the oral cavity could be sampled (being careful to maintain a central position in the mouth), and the mouth was then gently held closed around the probe during the measurement (30 s). The process was repeated for 3 measurements, after which an automatic average was generated. The 3 raw data points and the average were saved. Measurements were taken within 4 h after feeding.

### 2.5. Breath Scoring

On Days 0, 7, and 14 after the dog’s meal, a technician recorded each dog’s breath score [31,59] based on the following prompt and 10-point scale. Assessments were made within 4 h after feeding. The same technician performed all the assessments for all participants:

On a scale of 1–10, how do you rate the breath of the dog today? (1—No bad odor present, 5—Smelling the dog’s breath is unpleasant; some bad odor is present, 10—You cannot stand the smell of the dog’s breath).

1 / 2 / 3 / 4 / 5 / 6 / 7 / 8 / 9 / 10

### 2.6. Statistical Analysis

The use of one-tailed statistical tests was pre-determined based on trends observed in data collected from a preliminary internal study.

The following statistical analyses were performed on the VSC level data: A Shapiro-Wilke test was used to assess if the data were normally distributed. A one-tailed Wilcoxon test was performed on the average VSC level of each participant to assess how VSC levels changed from baseline within a group. A mixed linear regression was performed on the log-transformed data to assess any differences between the repeated measures of the two groups.

The following statistical analyses were performed on the breath score data: A Shapiro-Wilke test was used to assess if the data were normally distributed. A one-tailed paired Wilcoxon test was performed on the breath score of each participant to assess how malodor perception changed from baseline within a group. The absolute and relative change from baseline was calculated for each participant on Day 7 and Day 14. A one-tailed Mann–Whitney test on the absolute and relative change in breath score was used to assess any differences between the two groups.

## 3. Results

No adverse events were recorded, and no participants were removed from the study.

**Figure 1:**
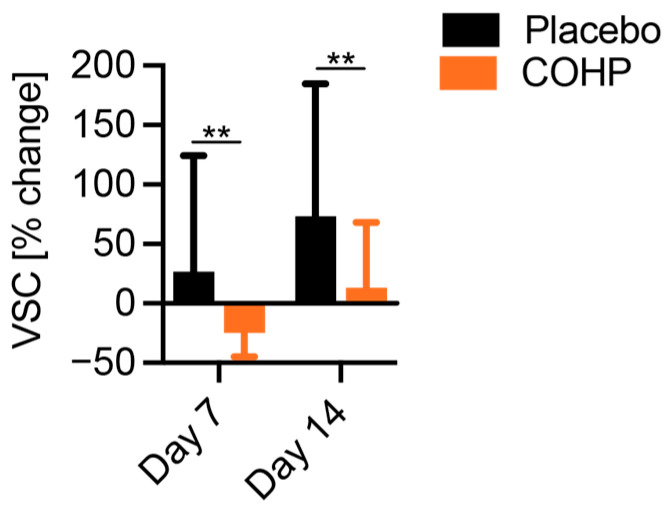
Relative changes in VSC levels. On Day 7 and Day 14, the change in VSCs from baseline was significantly lower in the COHP group compared to placebo (*p* = 0.004 at both timepoints, mixed linear regression of log-transformed data, ** indicates a *p*-value < 0.01). Bars indicate the mean and error bars indicate the standard deviation.

COHP effectively reduced the compounds responsible for bad breath within the duration of the study as assessed by the Halimeter (Table 1). In the placebo group, VSCs increased over the course of the study. There was a significant decrease in VSCs on Day 7 in the COHP group (median change = −22%; *p* = 0.002, one-tailed Wilcoxon test). The placebo group showed no change on Day 7 (*p* = 0.46). There was a significant increase in VSCs on Day 14 in the placebo group (median change = +35%; *p* = 0.039, one-tailed Wilcoxon test), while the COHP group showed no change on Day 14 (*p* = 0.4, one-tailed Wilcoxon test). Overall, COHP reduced VSC levels 27% compared to the placebo group on Days 7 and 14 (Figure 1, *p* = 0.004 at both timepoints, mixed linear regression of log-transformed data).

**Figure 2:**
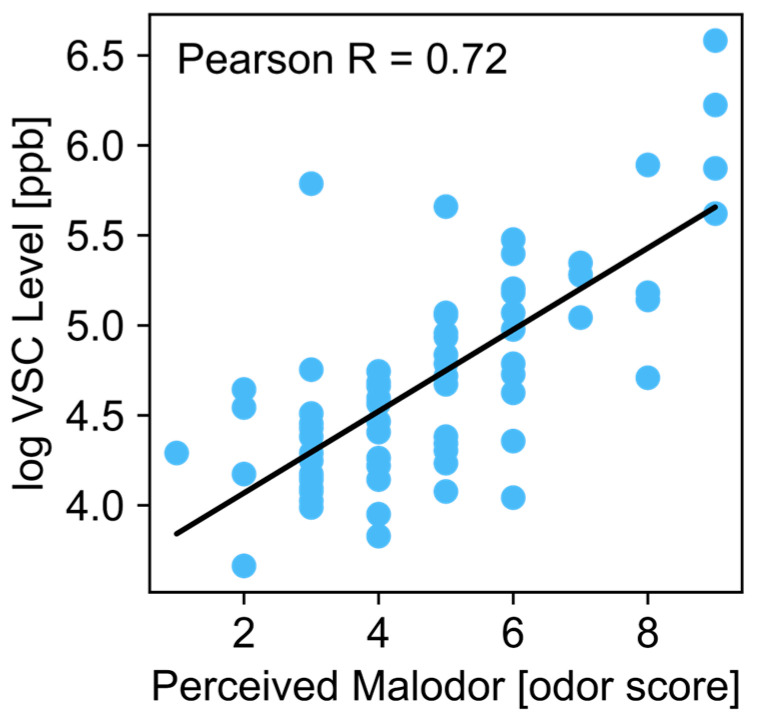
Correlation between perceived malodor and VSC level. The base-10 logarithmic transformation of measured VSCs correlated with perceived malodor (Pearson R coefficient of 0.72). Points represent measurements of single participants from both groups at a single timepoint. The line represents a linear fit of the data.

In addition to the reduction in VSCs in the COHP group there was a trend toward decreased perception of halitosis, and these data were consistent with the VSC data. At Day 7, in line with the VSC level data, COHP drove a decreasing trend in perceived malodor (median change = −10%, *p* = 0.21) whereas perceived malodor for the placebo group did not change (median change = 0%, *p* = 0.5, one-tailed paired Wilcoxon test). In addition, only 3 out of 12 dogs in the placebo group had an improved malodor score compared to Day 0, whereas 6 out of 12 dogs in the COHP group did (2× increase over the placebo). At Day 14 the placebo drove an increasing trend in perceived malodor (median change = +20%; *p* = 0.17, one-tailed paired Wilcoxon test), whereas perceived malodor for the COHP group did not change (median change = 0%; *p* = 0.29, one-tailed paired Wilcoxon test). Of note, the base-10 logarithmic transformation of VSC level was correlated with perceived malodor (Figure 2).

## 4. Discussion

Halitosis in dogs is highly prevalent; it not only poses challenges to relations between dogs and humans, it is also associated with periodontal disease [5,6,9,10,11]. Existing canine oral health solutions often focus on plaque and tartar without addressing halitosis directly [30,40,44,47,48]. Microbially derived solutions are particularly well-suited to target the pathogenic bacteria that cause halitosis and improve the fundamental microbial imbalances that contribute to poor oral health [49,50,51,52]. The purpose of this study was to investigate the ability of a novel postbiotic ingredient in reducing oral malodor in dogs.

We observed a significant reduction in VSC level on Day 7 in the COHP group in comparison to the baseline, and COHP fully prevented any increase in VSC level on Day 14, whereas the placebo group VSC level had increased by 35% from its baseline, likely due to the interruption in regular dental enrichment. An increase in VSCs was previously observed in a dirty tooth model study in which dental enrichment was withheld [60]. To understand how this performance compares to that of other oral health products, we compared our results to other studies of similar design. In one exemplary study by Carroll et al. on market-leading dental chews, an increase in VSCs was observed on Day 7, and on Day 14, the VSC level of the treatment group had increased and was about 50% of the control group [45]. These results indicate that COHP is roughly twice as effective as these dental chews. This comparison provides particularly compelling evidence of COHP’s efficacy given that COHP was delivered as a single-ingredient powder topper, whereas the dental chews combine mechanical action, the extended contact time of a chew, and many different oral health active ingredients. Beyond this, as noted by Carroll et al., most studies on dental chews have only reported effects that are ~10% to ~60% as large as what was observed in this study [35,36,45,46]. A more recent study by Wang et al. reports a comparable effect to that found by Carroll et al. on Day 29 [45,61]. Another recent study by Oba et al. also saw a significant decrease in VSCs compared to the placebo, but the increase in VSCs was modest in the placebo group, and treatment VSCs rose above baseline by Day 14 [37]. It is notable that the rapid onset of effects (within 7 days) and the sustained benefits observed through Day 14 in this study indicate that COHP could provide a practical and effective solution to a widespread health issue in the canine population.

The correlation between measured VSC levels and perceived malodor suggests that COHP could improve relations between dogs and humans by improving human perception of their pet’s breath [59,62]. While the changes to perceived malodor were not statistically significant, twice as many participants had improved odor scores in the COHP group compared to the placebo group at Day 7, suggesting that the postbiotic components may modulate human perception of canine oral malodor. This study was powered to assess changes to VSC levels and groups were stratified based on VSC levels. As such, a study with more participants or a design focused on assessing changes to human perception of malodor may be required to observe significant changes to odor score, which can be more variable than analytical measurements, particularly when scorers are not specifically trained in odor perception [63,64,65].

The primary focus of this study was halitosis, which we evaluated through two distinct types of measurement, VSC levels and breath scoring. While the study was conducted using colony animals, it aimed to reflect a real-world scenario focused on dogs with mild to medium halitosis that do not receive regular professional dental cleanings, by implementing a dirty tooth model. The use of a dirty tooth model also ensured there would be a measurable VSC signal throughout the study. In contrast to plaque and tartar focused studies, we did not assess a baseline plaque or gingivitis score, nor then could we stratify based on these variables. The absence of this additional gingival/plaque strata during randomization may, though not necessarily, introduce an imbalance between the groups, such as any uncontrolled variable would, because they are allocated at random between the groups. However, it is well established that the extent of halitosis is highly correlated with the extent of periodontal disease in both dogs and humans [3,10,11,66,67,68,69]. Due to this high correlation, it is expected that in studies that balance groups based on a halitosis-related variable, such as was carried out here using VSC levels, any imbalance in periodontal disease-related variables would be limited. The lack of assessment of plaque, tartar, and gingivitis remains a limitation of this study, and performing these assessments, together with utilizing larger cohorts, would allow further stratification by additional variables, such as ones related to the extent of periodontal disease or the breed, in future studies.

The effectiveness of COHP’s postbiotic approach provides valuable insights into managing canine oral health. Postbiotics show promising results in improving oral health in humans, but evidence in dogs is limited to other health benefits, such as improvements to gut and immune health [70,71,72,73,74,75,76]. In two separate studies, two different oral treatments consisting of postbiotics from two-three different strains had positive effects on the human oral microbiota and increased markers associated with oral immune health, such as salivary IgA [51,77]. Additionally, postbiotics derived from multi-species oral microbiome samples have been found to mitigate chemotherapy-induced oral dysbiosis [50].

COHP’s ability to significantly reduce VSCs validates the mechanistic approach of addressing the underlying microbial causes of oral malodor, rather than masking symptoms; COHP achieves measurable improvements in a key indicator of oral health. Increase in VSCs is a crucial mechanism in the progression of periodontal disease, as VSCs, and, in particular, H_2_S, have direct cytotoxic effects on oral tissues [13]. Consistent with other studies, the fact that VSC levels increased in the placebo group over the study period highlights the importance of effective preventative measures [37,45].

## 5. Conclusions

This clinical study demonstrated COHP’s ability to reduce the compounds that underlie halitosis in dogs. COHP significantly reduced VSCs by 27% compared to the placebo throughout the study, and significantly reduced VSCs after only 7 days of treatment. Effectively, COHP fully prevented any increase in halitosis. Additionally, measured VSCs were positively correlated with an increased human perception of malodor and twice as many participants in the COHP group had improved odor scores at Day 7, suggesting that COHP can improve the human perception of their pet’s breath. Together, these findings validate the efficacy of our novel postbiotic in reducing canine halitosis.

## Figures and Tables

**Table 1:** Group VSC levels in parts per billion (mean ± std) throughout the study.

	Timepoint		
Group	Day 0	Day 7	Day 14
Placebo	119.4 ± 61	154.3 ± 148	207.9 ± 190
COHP	119.4 ± 48	90.0 ± 46	126.8 ± 56

## Data Availability

Datasets available upon reasonable request from the authors.
